# Enhanced recovery after microdiscectomy: reductions in opioid use, length of stay and cost

**DOI:** 10.1186/s12893-023-02130-3

**Published:** 2023-08-29

**Authors:** Yun Lu, Jiang Long, Xue Leng, Yaqing Zhang, Guanzhong Wang, Jiawei Yuan, Libangxi Liu, Jiawei Fu, Minghui Yang, Yu Chen, Changqing Li, Yue Zhou, Chencheng Feng, Bo Huang

**Affiliations:** 1 Department of Orthopedics Xinqiao Hospital, Army Medical University, 183 Xinqiao Main Street, Shapingba, Chongqing, 400037 People’s Republic of China; 2 Department of Spine surgery, Sixth Affiliated Hospital of Xinjiang Medical University, 39 Wuxing South Road, Tianshan District, Urumqi, Xinjiang, 830002 People’s Republic of China

**Keywords:** Enhanced recovery after surgery, Microdiscectomy, Length of stay, Cost, Opioid use

## Abstract

**Background:**

Enhanced recovery after surgery (ERAS) protocols are widely used worldwide. Recently, studies of the ERAS program in spinal surgery subspecialties have been reported. The aim of this study was to evaluate the impacts of ERAS in minimally invasive microdiscectomy (MD) surgery.

**Methods:**

This was a retrospective cohort study of patients undergoing MD at a single center. From March 2018 to March 2021, 286 patients were in the ERAS group. A total of 140 patients from March 2017 to February 2018 were in the conventional group. The outcomes included length of stay (LOS), the postoperative numeric rating scale (NRS), complications, 30-day readmission rate, 30-day reoperation rate and cost. Moreover, perioperative factors were also evaluated.

**Results:**

Compared with the conventional group, the LOS and cost were reduced in the ERAS group. There were no significant differences in the NRS, complication rate, 30-day readmission or reoperation rates between the groups. Furthermore, postoperative drainage volume, and postoperative opioid use were lower in the ERAS group.

**Conclusions:**

The ERAS protocol for MD surgery reduces LOS, cost and opioid use and accelerates patient recovery.

## Background

Enhanced recovery after surgery (ERAS) was first introduced in the late 1990s by Henrik Kehlet. The aim of ERAS is to minimize the surgical stress response and reduce surgical complications while accelerating recovery [1]. The core principles of ERAS include (1) a patient-focused surgical journey, (2) multidisciplinary care, (3) multimodal analgesia, and (4) evidence-based practice [2]. Currently, ERAS has been successfully implemented in many surgical specialties, including colorectal surgery, general surgery, thoracic surgery, urology and gynecology. It has been shown to be effective and safe in improving patient outcomes [3]. Recently, spinal surgery practices have only begun applying ERAS protocols, and these spinal surgeries include anterior cervical discectomy and fusion, lumbar fusion, idiopathic scoliosis surgery and spinal oncology [4–7].

Discectomy is a classic surgical technique for the treatment of lumbar disc herniation (LDH). Traditional open discectomy causes disruption of the paraspinal muscles. Moreover, some complications are associated with discectomy, including durotomy, nerve root injury, surgical site infection and epidural hematoma. With the development of minimally invasive spine surgery techniques, the operating microscope and tubular or expanding retractor systems have been applied to discectomy surgery, thus introducing the concept of microdiscectomy (MD). Compared with open discectomy, MD has a more minimally invasive operation and can avoid the detachment of the lumbar paraspinous muscles. MD has become a standardized surgery and has been widely used for the treatment of LDH [8]. However, the length of stay (LOS), cost, complications and postoperative pain of MD vary widely. An ERAS protocol tailored for MD is required to improve perioperative surgical outcomes.

Herein, we established and implemented an ERAS protocol for MD. We aimed to compare the surgical outcomes of patients treated with MD before and after ERAS protocol implementation.

## Methods

### Study Design

This study was approved by the ethics committee of Xinqiao Hospital of Army Medical University and the IRB approval number is 2022-R.No.380-01. All methods were carried out in accordance with relevant guidelines and regulations. A historically controlled study was designed to evaluate the influence of the ERAS protocol on patients undergoing microdiscectomy. All surgeries were performed by the same surgeon (Dr. Huang and Dr. Zhang) with extensive experience in MIS technique. The retrospective analysis included two cohorts before (conventional group) and after (ERAS group) ERAS protocol implementation in March 2018. The data collection started in March 2017 and ended in March 2021. The inclusion criteria included patients who underwent single-level MD. The exclusion criteria were as follows: ① patients undergoing multilevel MD surgery and ② patients with a history of spinal surgery, spinal infection, neoplasm and deformity. Data collection was performed by manual review of the electronic medical record system, so all patients who met exclusion criteria were not included in the analysis. Informed consent was waived per the study design by the ethics committee of Xinqiao Hospital of Army Medical University.

### ERAS pathway

**Fig. 1:**
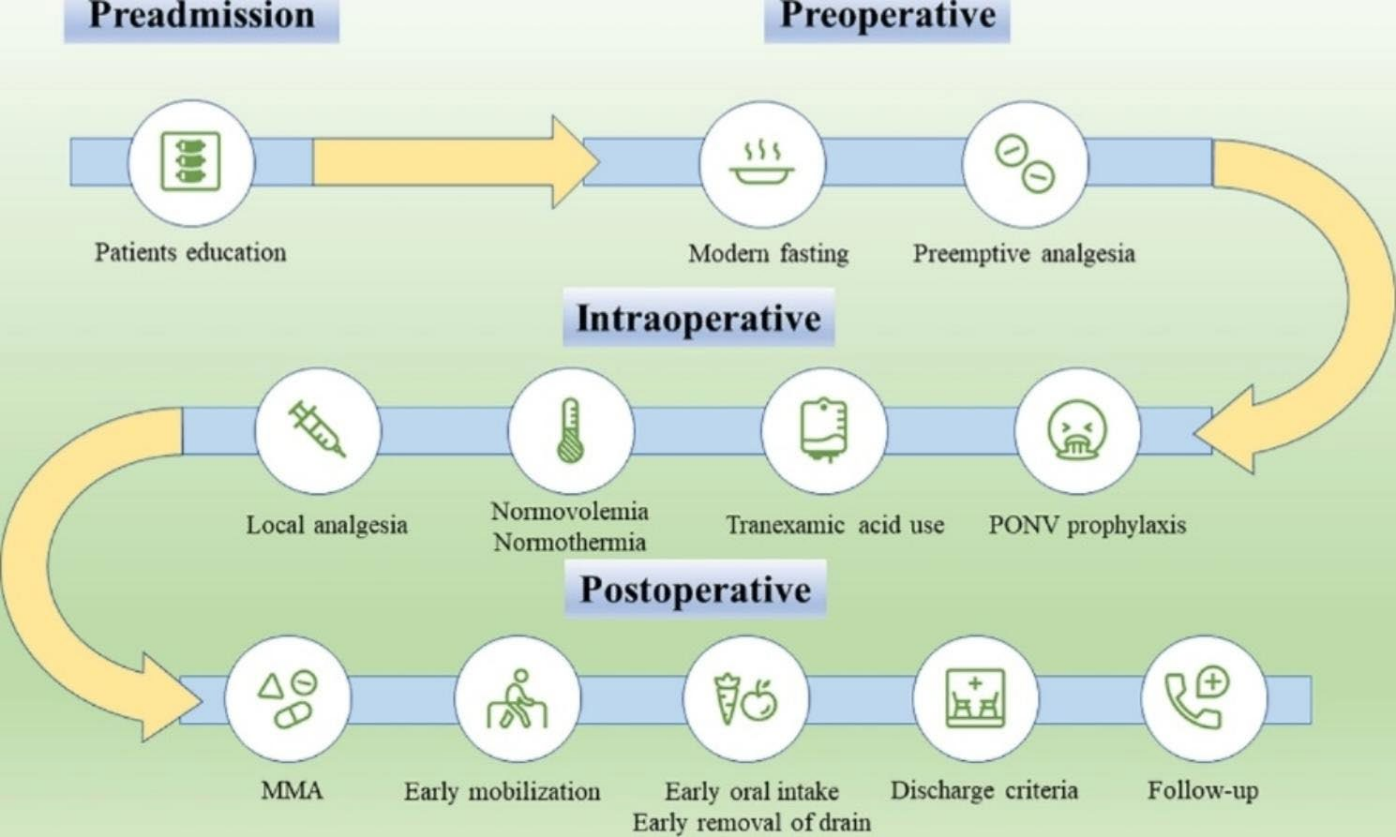
The components of ERAS protocol Abbreviations: PONV, postoperative nausea and vomiting; MMA, multimodal analgesia

Our ERAS protocol was designed based on the patient’s journey through MD. The multidisciplinary ERAS team consisted of practitioners from spinal surgery, anesthesiology, nursing, nutrition, clinical pharmacy, rehabilitation and hospital administrators. The ERAS protocol included four chronological phases: preadmission, preoperative, intraoperative, and postoperative phases (Fig. 1). The detailed components are shown in Table 1.

**Table 1:** Components of the ERAS protocol and conventional protocol

Phases	Components	ERAS Protocol	Conventional Protocol	Reference
**Preadmission**	Patients education	• Expectation setting • ERAS education regarding the surgical technique, rehabilitation, pain management, discharge and follow-up plan	Routine informed consent	[29]
**Preoperative**	Modern fasting	• Solids until 6 h and carbohydrate beverages until 2 h before surgery	Fasting 8 h	[30]
	Preemptive analgesia	• Oral preemptive analgesia (200 mg celecoxib and 75 mg pregabalin) provided in the holding area on the day of surgery	Not routinely used	[9–11]
**Intraoperative**	PONV prophylaxis	• 10 mg dexamethasone administrated during anesthetic induction, 0.3 mg ramosetron given 30 min before the end of surgery	No routinely administrated	[31]
	Tranexamic acid use	• 1 g bolus 30 min before incision followed by an infusion of 0.5 g/hour	Not routinely used	[18]
	Normovolemia	• Goal-directed fluid management	Caregiver preference	[32]
	Normothermia	• Achieving normothermia at 36℃ by using convective warming devices	Performed using blankets	[33]
	Foley catheter	• No Foley catheter utilized	Catheterization before anesthesia	[34]
	MIS techniques	• Microscope assisted surgery via tubular retractors	No microscope	[2, 35]
	Local analgesia	• Local infiltration analgesia at the end of surgery	Rarely used	[35]
**Postoperative**	MMA	• Opioid sparing, multimodal regimen	Caregiver preference	[35]
	Early mobilization	• Handouts including mobilization methods and goals provided by practitioners, patients encouraged to ambulate after anesthetic resuscitation	Not provided handouts, patients required to have bed rest on POD 1–3	[35–37]
	Early oral intake	• oral diet after recovery from anesthesia	Not provided clear liquids	[35]
	Early removal of drain	• POD 1	Based on personal preference	[35]
	Discharge criteria	• Activities of daily living without help, adequate pain control (NRS < 3), no complications or appropriate management of complications	Caregiver preference	[38]
	Follow-up	• A mobile app was used for real-time follow-up	Periodic outpatient follow-up	[36]

ERAS, enhanced recovery after surgery; PONV, postoperative nausea and vomiting; MIS, minimally invasive surgery; MMA, multimodal analgesia; POD, postoperative day; NRS, Numerical Rating Scale

During the preadmission phase, the patient education included expectation setting, which determines the patient’s own initiative in treatment and rehabilitation. At the same time, we provided ERAS education regarding the surgical technique, recovery goals, pain management, discharge criteria, and follow-up plans.

Preoperatively, the patients were permitted to take solids until 6 h and carbohydrate beverages until 2 h before surgery for modern fasting. For preemptive analgesia, 200 mg celecoxib and 75 mg pregabalin were administered orally 1 h before surgery in the holding area [9–11].

Before the surgical incision was made, the patients were given 1 g of tranexamic acid intravenously followed by an infusion of 0.5 g/hour. The surgery was assisted by microscopy via tubular retractors. The surgery was performed under general anesthesia. And the anesthesia team used the same induction and intraoperative pain regimen as the conventional group. Intraoperatively, aiming to maintain normovolemia and normothermia, goal-directed fluid management and convective warming devices were adopted. In addition, 10 mg dexamethasone was administered during anesthetic induction and 0.3 mg ramosetron was given 30 min before the end of surgery for postoperative nausea and vomiting (PONV) prophylaxis. At the end of the surgery, the incision was anesthetized with ropivacaine hydrochloride (5 mg/mL).

Postoperatively, multimodal pain management was provided (celecoxib 200 mg and pregabalin 75 mg were given every 12 h. If pain was poorly controlled, tramadol 100 mg was administered intramuscularly). Handouts including mobilization methods and rehabilitation goals were provided. An oral diet was permissible after recovery from anesthesia. Furthermore, the drain was removed on postoperative day 1 (POD 1), and the patients were encouraged to ambulate after anesthetic resuscitation. The discharge criteria were clearly defined. After discharge, a mobile app was used for follow-up.

### Outcomes

The outcomes included length of stay (LOS), financial cost to the hospital, postoperative numeric rating scale (NRS), 30-day readmission rate, 30-day reoperation rate, and complications. In terms of financial cost to the hospital, including drugs, tests, examinations, treatment, surgery, anesthesia, materials, beds, and nursing care. Furthermore, we reviewed the perioperative factors including blood loss, surgical drainage, and opioid use.

### Statistical analysis

Continuous variables are summarized as the mean (standard deviation) and median (IQR). Categorical variables were summarized with frequencies and percentages. We used the Levene test to test for homogeneity of variance. For comparison, we used independent sample t test or rank sum test for the continuous variables and χ^2^ test or Fisher exact test for the categorical variables. Statistical analysis was performed with SPSS (version 25.0 [IBM Corp., Armonk, New York, USA]). P < 0.05 was considered statistically significant.

## Results

### Patient characteristics

A total of 426 patients were in this study: 140 patients in the conventional group and 286 patients in the ERAS group. There were no significant differences between the two groups in terms of demographics, comorbidities, radiological manifestation, operation level or American Society of Anesthesiologists (ASA) grade (P > 0.05, Table 2).

**Table 2:** Demographic and Baseline Characteristics of Patients

Parameter	Convention (n = 140)	ERAS (n = 286)	p value	
Age (years), median (IQR)	48.0 (40.0,60.0)	47.5 (37.0,59.0)	0.679	
Gender (male/female)	74/66	170/116	0.197	
BMI (kg/m^2^), median (IQR)	24.2 (21.8,26.4)	24.0 (21.8,26.7)	0.964	
Radiological manifestation (n)				
-lumbar disc herniation	140	286	1.000	
-lumbar spinal stenosis	83	154	0.288	
-lumbar spondylolisthesis	4	11	0.603	
-ligamentum flavum hypertrophy	26	54	0.939	
-lumbar facet arthritis	79	150	0.439	
The operation level (n)			0.728	
-L3/L4	3	4		
-L4/L5	86	169		
-L5/S1	51	113		
Diabetes mellitus (n)	7	19	0.506	
Hypertension (n)	22	29	0.096	
Chronic cardiovascular disease (n)	1	2	0.986	
Rheumatoid arthritis (n)	0	1	0.484	
Chronic bronchitis (n)	0	2	0.321	
Chronic nephritis (n)	0	1	0.484	
SLE (n)	0	1	0.484	
Bronchial Asthma (n)	1	0	0.152	
ASA grade (n)			0.077	
-ASA 1 -ASA 2 -ASA 3	0 118 22	10 236 40		

IQR, interquartile range; BMI, Body mass index; SLE, Systemic Lupus Erythematosus; ASA, American Society of Anesthesiologists

### Outcome Metrics

The ERAS group showed a significantly shorter LOS than the conventional group. Meanwhile, the cost was dramatically reduced in the ERAS group. With respect to perioperative factors, the ERAS group had less postoperative drainage than the conventional group. Moreover, there were no significant differences in the NRS scores at POD 0–3. The postoperative opioid use rate in the ERAS group was significantly decreased compared with that in the conventional group.

The complication rate of the conventional group was 2.9%, including 2 cases of durotomy and 1 case of surgical site infection. One case involved an epidural hematoma with radiculopathy after surgery, and the patient underwent readmission and reoperation. The complication rate in the ERAS group was 2.8%, including 5 cases of durotomy and 1 case of surgical incision infection. And two patients were rehospitalized and reoperated due to epidural hematoma with radiculopathy. Furthermore, one patient in the ERAS group required readmission due to recurrent LDH. There were no significant differences in complications, 30-day readmission or the reoperation rates between the two groups (P > 0.05, Table 3).

**Table 3:** Perioperative Factors and Postoperative Outcomes

Parameter	Convention (n = 140)	ERAS (n = 286)	p value
LOS, median (IQR)	4 (3,4)	3 (2,4)	<0.001
Intraoperative blood loss (ml), median (IQR)	50 (20,100)	50 (30,100)	0.338
Postoperative drainage (ml), median (IQR)	20 (10,35)	15 (8,28)	<0.001
Cost (yuan), median (IQR)	22,795 (20,943, 24,600)	19,424 (18,068, 21,169)	<0.001
Opioid use, n (rate)	17 (12.1%)	12 (4.2%)	0.002
Overall complications, n (rate)	4 (2.9%)	8 (2.8%)	0.972
Durotomy, n (rate)	2 (1.4%)	5 (1.7%)	0.807
Surgical site infection, n (rate)	1 (0.7%)	1 (0.3%)	0.605
Epidural hematoma with radiculopathy, n (rate)	1 (0.7%)	2 (0.7%)	0.986
Postoperative NRS, median (IQR)			
POD 0	2 (1.00,2.00)	2 (1.00,2.00)	0.936
POD 1	2 (2.00,2.00)	2 (2.00,2.00)	0.750
POD 2	2 (1.75,2.00)	2 (2.00,2.00)	0.371
POD 3	2 (1.00,2.00)	2 (1.00,2.00)	0.683
30-day readmission, n (rate)	1 (0.7%)	3 (1.0%)	0.737
30-day reoperation, n (rate)	1 (0.7%)	2 (0.7%)	0.986

LOS, Length of Stay; NRS, Numeric rating scale; POD, Postoperative day

## Discussion

In the current study, the implementation of the ERAS protocol resulted in decreased LOS and cost without increasing the complication, 30-day readmission and reoperation rates. Compared with the conventional group, postoperative drainage, and opioid use were lower in the ERAS group.

Few studies have reported the implementation of ERAS on MD and clinical outcomes. In 2018, a study reported that the ERAS pathway leads to short LOS, minimal complications and no 90-day readmission [12]. Ebru et al. investigated the effects of ERAS on single-level lumbar MD. They found that the introduction of the ERAS protocol was associated with shorter LOS, less blood loss and operation time, earlier oral intake and mobilization, and lower cost [13]. In a retrospective paired study, 386 patients who underwent spinal surgery, including MD surgery, were included and assessed. The results showed that the ERAS protocol reduced LOS without additional adverse events, including postoperative pain, complications, and readmissions [14]. Herein, we established and implemented a tailored ERAS pathway for MD. The results of this study further reveal the effects of ERAS on MD surgery, and consequently provide evidence for the application of ERAS in spinal surgery.

There are numerous factors affecting LOS, including comorbidities, perioperative interventions, postoperative pain, complications, and the time of ambulation. In our ERAS protocol, we emphasized the importance of early ambulation. We encouraged patients to start ambulation under the guidance of a nurse after recovering from anesthesia. A previous study reported that early ambulation reduces perioperative complications and shortens LOS by 34% [15]. On the other hand, urinary catheterization has some adverse effects on patients, including urethral pain, urinary tract infection, urethral trauma, bladder stones and urinary retention [16]. All these complications might limit the postoperative activities of patients and impede early discharge. Thus, we did not utilize urinary catheterization and encouraged urination before anesthesia. Goal-directed fluid management was also applied to control intraoperative infusion.

In fact, after ERAS protocol implementation, the LOS was still relatively high. There was a study reporting an ERAS protocol for lumbar microdiscectomy surgery based on outpatient surgery, and the result showed that the median LOS was 279 min [12]. With the development of modern surgical and anesthetic technologies, the medical care model requiring long-term hospitalization has great challenges. Nonetheless, in the absence of evidence, people still have great concerns about a new concept of medical care [17]. As a key target of ERAS pathway implementation, there are several difficulties faced while attempting to reduce the LOS, and the difficulties include health care resources, local medical policies, and traditional concepts of patients and caregivers. Based on the traditional concept of patients in China, a longer LOS means better care and recovery. If patients are discharged early, they will feel insecure and anxious. Furthermore, it is difficult for patients to obtain high-quality medical services in the communities of China. Thus, we did not perform outpatient surgery for MD.

Tranexamic acid has been reported to reduce total blood loss and transfusion in spine surgery [18]. It was a crucial component in our MD-ERAS protocol. Noticeably, there was no significant difference in intraoperative blood loss between the conventional group and the ERAS group, which may be due to the minimally invasive nature of MD surgery. The minimal blood loss during MD did not reflect the benefits of tranexamic acid use. However, the postoperative drainage of the ERAS group was significantly less than that of the conventional group, which was probably attributed to the use of tranexamic acid [19]. Until now, the use of drainage in spine surgery has remained controversial. Wound drainage is intended to prevent the formation of epidural hematoma and wound-related complications. However, it can also cause retrograde infection and increase postoperative blood loss [20]. In our center, drainage is routinely placed to reduce the potential risks of epidural hematoma, lower back discomfort, and surgical site infection [21]. Furthermore, the drainage was removed at POD 1 to minimize the restriction on the postoperative activities of patients.

If chronic pain persists after spinal surgery, some patients may develop post-laminectomy syndrome [22]. Therefore, postoperative analgesia is an important component of the ERAS pathway for spine surgery. The commonly used analgesic regimens include opioids and nonopioids. Opioids have some adverse effects on patients, including somnolence, ileus, urinary retention, respiratory depression and bone fracture, which may delay the recovery of patients and extend LOS [23]. A previous study through a subgroup analysis of patients with LOS < 3 and LOS > 3 found that patients with LOS < 3 had a significant reduction in opioid use at POD 2, indicating the influence of opioid use on LOS [24]. In contrast, nonopioid analgesics effectively control postoperative pain with fewer adverse effects [25]. Moreover, a multimodal approach of pain management has been shown to effectively reduce postoperative pain in spine surgery [26]. For all these reasons, we adopted multimodal analgesia (MMA) with opioid sparing in the ERAS protocol. Compared with the conventional group, a significant reduction in the opioid use rate was observed in the ERAS group, although there was no significant difference in the postoperative NRS between the two groups. However, the reduction in opioid use has the potential benefit of reducing opioid-related side effects [23].

The complications of discectomy include durotomy, epidural hematoma, nerve root injury and surgical site infection [8, 27]. In the current study, there was not a significant difference in the rate of complications between the two groups. Furthermore, there were no significant differences in the 30-day readmission and reoperation rates between the two groups. The results were consistent with previous studies. Soffin et al. found that patients treated with the ERAS protocol had no readmission or reoperation within 90 days of MD surgery due to complications [12]. Another study also reported 5 years of experience with the ERAS protocol for MD. The results showed that the rate of 30-day readmissions was 0.62% [28]. In conclusion, the implementation of the ERAS protocol in MD surgery was demonstrated to be safe. On the other hand, the hospitalization cost of the ERAS group was reduced significantly, indicating that the ERAS approach tailored for MD is cost-effective.

This study has several shortcomings. First, it was a retrospective analysis without randomization or blinding. The two groups were in different time frames. Therefore, recall bias and selection bias were inevitable. The strength of evidence was restricted. To further confirm the efficacy and safety of ERAS for MD, randomized controlled trials will be required to provide more evidence at higher levels. Second, the protocol was designed by our multidisciplinary ERAS team consisting of practitioners from spinal surgery, anesthesiology, nursing, nutrition, clinical pharmacy, rehabilitation and hospital administrators based on evidence-based medical evidence. However, the data did not enroll in ENCARE. Third, in terms of costs, in addition to hospital costs, other costs such as the new HR recruitment needed, ERAS enrolment and training, development of app should be considered if they exist, although they are difficult to calculate. Fourth, the long-term effects of ERAS on the Oswestry Disability Index and patient satisfaction were unknown. Long-term follow-up should be carried out in further studies.

## Conclusions

The ERAS protocol reduces the LOS, cost, postoperative drainage, and opioid use of patients undergoing MD surgery without causing adverse events, including complications, readmissions and reoperations. ERAS is a cost-effective pathway for promoting rapid recovery of patients from MD.

## Data Availability

All data are fully available without restriction. The database used in this study is available from the corresponding author on reasonable request.

## Abbreviations

ERAS: Enhanced recovery after surgery
MD: Microdiscectomy
LOS: Length of stay
NRS: Numeric rating scale
LDH: Lumbar disc herniation
PONV: Postoperative nausea and vomiting
MMA: Multimodal analgesia
MIS: Minimally invasive surgery
POD: Postoperative day
ASA: American Society of Anesthesiologists
IQR: Interquartile range
BMI: Body mass index
SLE: Systemic Lupus Erythematosus
